# Topiramate Enhances GABAergic Tone to Orexigenic Neuropeptide Y/Agouti‐Related Peptide (NPY/AgRP) Neurons

**DOI:** 10.1002/oby.70051

**Published:** 2025-10-02

**Authors:** Moein Minbashi Moeini, Olivier Lavoie, Alexandre Caron, Kevin W. Williams, Natalie J. Michael

**Affiliations:** ^1^ Faculty of Pharmacy Université Laval Québec Québec Canada; ^2^ Québec Heart and Lung Research Institute Québec Québec Canada; ^3^ Center for Hypothalamic Research, Department of Internal Medicine, Peter O’Donnell Jr. Brain Institute, UT Southwestern Medical Center Dallas Texas USA

**Keywords:** hypothalamus, melanocortin system, neurometabolism, neuropeptide Y neurons, topiramate

## Abstract

**Objective:**

Topiramate is a medication used off‐label, or in combination with phentermine, for the management of obesity. However, its mechanism of action remains elusive. As many obesity medications target the brain, we aimed to determine if topiramate influences the activity of hypothalamic melanocortin neurons known to regulate energy balance.

**Methods:**

Transgenic mice expressing a fluorescent protein in either “orexigenic” neuropeptide Y/agouti‐related peptide (NPY/AgRP) or “anorexigenic” pro‐opiomelanocortin (POMC) neurons were used to perform whole‐cell patch clamp electrophysiology experiments in the arcuate nucleus (ARC) of the hypothalamus.

**Results:**

Topiramate (1 μM) strongly inhibited NPY/AgRP neuron electrical excitability. Despite topiramate's well‐known actions at GABA_A_ receptors, we demonstrate that the topiramate‐induced inhibition of NPY/AgRP neurons does not involve GABA_A_ receptors. The effects of topiramate on NPY/AgRP neurons were suppressed by inhibitors of synaptic transmission and after blockade of GABA_B_ receptors or potassium channels. In contrast, topiramate had negligible influence on the activity of POMC neurons.

**Conclusions:**

This study is the first demonstration that topiramate strongly inhibits the activity of ARC NPY/AgRP neurons and suggests that enhanced GABAergic tone to these neurons mediates this effect. The ability of topiramate to inhibit the orexigenic NPY/AgRP neurons may underlie some of its weight‐lowering properties.

## 1 Introduction

Pharmacotherapy is an essential component in the treatment and long‐term management of obesity [1]. Multiple antiobesity medications (AOMs) are effective in reducing body weight and improving cardiometabolic parameters, especially when combined with behavioral and lifestyle interventions [2]. In recent years, the number of AOMs approved or in development for the treatment of obesity has increased. Most AOMs act on the central nervous system, with the brain playing a central role in mediating their weight‐lowering properties [3, 4]. Despite AOMs being able to access multiple brain regions [5], the exact cellular and molecular mechanisms underlying their weight‐lowering effects are still being uncovered. Elucidation of the precise neurobiological mechanisms of action of AOMs may enable the development of more precise molecules, additional combination therapies, or greater treatment effectiveness by aligning the type of AOMs prescribed with individual patient characteristics.

Topiramate is a prime example of a medication with significant metabolic benefits despite an incomplete understanding of its mechanisms of action. Originally prescribed as an antiepileptic agent, topiramate was later found to promote weight loss [6, 7, 8], which saw it used off‐label for the treatment of obesity [9]. Studies in humans have demonstrated that it reduces food intake and leads to an average weight loss of approximately 5% of initial body weight [10], with even greater reductions (~15%) when combined with diet and lifestyle interventions [11]. Supporting its efficacy in reducing body weight, in 2012, topiramate was approved by the Food and Drug Administration (FDA) as a combination therapy for the treatment of obesity (phentermine/topiramate) [12]. Despite its effectiveness, the neurobiological mechanisms underlying these effects remain poorly defined.

Several hypotheses have been proposed to explain how topiramate induces weight loss. These range from decreases in food intake, increased satiety, effects on the reward system, and alterations in the metabolism of sugar and lipids [13, 14]. However, these proposed mechanisms do not consider cellular and molecular mediators of the effects. Several different receptors and ion channels have also been proposed as targets of topiramate. This includes GABA_A_ receptors, voltage‐gated sodium channels, high‐voltage‐activated calcium channels, and AMPA/kainate receptors [15, 16]. Furthermore, topiramate increases GABA levels in the brain [17], suggesting it may alter neuronal excitability and function through multiple mechanisms.

Of the brain regions regulating energy balance, the hypothalamus plays a pivotal role in sensing and integrating metabolic signals to regulate feeding behavior and body weight. In particular, melanocortin neurons located in the arcuate nucleus (ARC) of the hypothalamus are key components contributing to the maintenance of energy homeostasis [18, 19]. This includes “orexigenic” neurons coexpressing neuropeptide Y and agouti‐related peptide (NPY/AgRP) and “anorexigenic” neurons expressing pro‐opiomelanocortin (POMC), which have opposing actions at downstream targets [19]. Importantly, many current or past AOMs influence hypothalamic metabolic pathways, including the central melanocortin system [3, 20]. However, whether topiramate also induces weight loss by targeting the melanocortin system remains to be determined.

Previous studies have assessed the effects of topiramate on *Npy* and *Pomc* gene expression within the hypothalamus. It has been shown that the expression of both *Npy* and *Pomc* increases following topiramate treatment; however, contradicting reports suggest that the gene expression of these neuropeptides does not change [13, 21, 22]. This highlights the need for more mechanistic studies that move beyond gene expression to explore functional neuronal activity. Therefore, in the present study, we investigated whether topiramate modulates the electrical excitability of hypothalamic melanocortin (NPY/AgRP and POMC) neurons known to regulate energy balance.

## 2 Methods

### 2.1 Animals

All experiments were conducted in accordance with National (Canadian and American) guidelines and were approved by either the Animal Care Committee of Université Laval (CPAUL) or the Institutional Animal Care and Use Committee (IACUC) of the University of Texas Southwestern Medical Center. Mice were maintained on a 12‐h light/dark cycle in temperature‐controlled facilities with free access to food and water. Mice were fed a standard chow diet (Teklad Rodent Diet, 16%–18% protein).

Transgenic mice were used to target genetically identified melanocortin neurons for electrophysiological recordings. This included *Npy*‐hrGFP mice (JAX stock #006417), which express a humanized Renilla green fluorescent protein (hrGFP) under the control of the mouse *Npy* promoter [23]. *Npy* expression was used as a surrogate marker for the identification of NPY/AgRP neurons, as done by others [24, 25, 26], although the coexpression of *Agrp* was not determined. The *Npy*‐hrGFP mice were also crossed with leptin receptor (*Lepr*)‐Cre mice [27] (JAX stock #032457) and tdTomato reporter mice (Ai14, JAX stock #007914) to generate *Npy*‐hrGFP::*Lepr*‐Cre::tdTomato (NLT) mice, allowing identification of *Lepr*‐expressing NPY neurons, as done previously [20]. *Pomc*‐CreERT2 mice [28] (RRID:MGI:5569339) were also bred with tdTomato reporter mice (Ai14, JAX stock #007914) to generate mice in which a red fluorescent protein was exclusively expressed in POMC neurons following a single dose of tamoxifen. Tamoxifen (0.15 mg/g; Sigma‐Aldrich, T5648) was suspended in corn oil (Sigma‐Aldrich, C8267), filtered (Sarstedt, Filtropur S 0.45 μm) and administered at 7–8 weeks of age via an intraperitoneal injection.

### 2.2 Electrophysiology

#### 2.2.1 Brain Slice Preparation

Whole‐cell patch clamp recordings were generated from NPY/AgRP and POMC neurons following preparation of coronal hypothalamic brain slices, as done previously [20, 29]. All mice were sacrificed between 8 and 12 weeks of age. Briefly, mice were anesthetized using isoflurane or chloral hydrate and decapitated, and the brain was rapidly removed and maintained in a modified sucrose‐based, ice‐cold (< 4°C) artificial cerebrospinal fluid (aCSF) containing (in mM): 213 sucrose, 2.5 KCl, 5 MgCl_2_, 1 CaCl_2_, 1 NaH_2_PO_4_, 26 NaHCO_3_, and 10 d‐glucose, continuously oxygenated with 95% O_2_ and 5% CO_2_.

Coronal brain slices (250 μm) containing the ARC were prepared using a vibratome (Leica VT1000S) and incubated (~32°C) for a minimum of 40 min in a standard aCSF containing (in mM): 126 NaCl, 2.8 KCl, 2.5 CaCl_2_, 1.25 NaH_2_PO_4_, 26 NaHCO_3_, 1.2 MgSO_4_, and 10 d‐glucose. Slices were then transferred to the recording chamber where they were continuously perfused with a heated, reduced d‐glucose concentration (5 mM) version of the standard aCSF.

#### 2.2.2 Whole‐Cell Patch Clamp Recordings

Whole‐cell patch clamp recordings were established from fluorescently labeled (NPY/AgRP and POMC) neurons using a Zeiss fixed stage microscope (Axio Examiner or Axioskop FS2) fitted with infrared video‐microscopy and fluorescence. Whole‐cell recordings were performed using an Axopatch 200B or Multiclamp 700B amplifier and Digidata (1550B or 1440A) digitizer (Molecular Devices). Current clamp data were filtered at 4 kHz. All data were recorded and stored on a personal computer for offline analysis with pClamp10 software (Molecular Devices).

Patch pipettes were pulled from thin‐walled borosilicate glass (TW150F‐4, World Precision Instruments) and had a resistance of approximately 5 MΩ when filled with an intracellular solution containing (in mM): 120 K‐gluconate, 10 KCl, 1 NaCl, 1 MgCl_2_, 1 CaCl_2_, 5 EGTA, 10 HEPES, and 2 Mg_2_ATP, adjusted for pH and osmolality with KOH and sucrose. All neurons were allowed to settle before the assessment of standard measures of electrical excitability.

### 2.3 Receptor Pharmaceutical Agents

All drugs were prepared as concentrated stocks, aliquoted, and stored < 4°C. Stock solutions were diluted to the required concentration in aCSF immediately prior to use and delivered to the recording chamber by a peristaltic perfusion system. All drugs were reconstituted in distilled water except for topiramate, picrotoxin, and CGP 54626 hydrochloride, which required the use of ethanol as the solvent. Topiramate, CNQX disodium salt, D‐AP5, tetrodotoxin (TTX), CGP 54626 hydrochloride, and picrotoxin were purchased from Tocris, and barium chloride (BaCl_2_) from Sigma.

### 2.4 Statistical Analysis

Comparisons between conditions were performed using two‐tailed paired *t*‐tests or repeated measures one‐way ANOVA using GraphPad Prism 10. Parametric statistics were used when data displayed a Gaussian distribution, and nonparametric statistics were used when this assumption was not met. The reported *p* values were adjusted for multiple comparisons. Data are presented as means ± SEM.

## 3 Results

### 3.1 Topiramate Strongly Inhibits ARC NPY/AgRP Neurons

**FIGURE 1:**
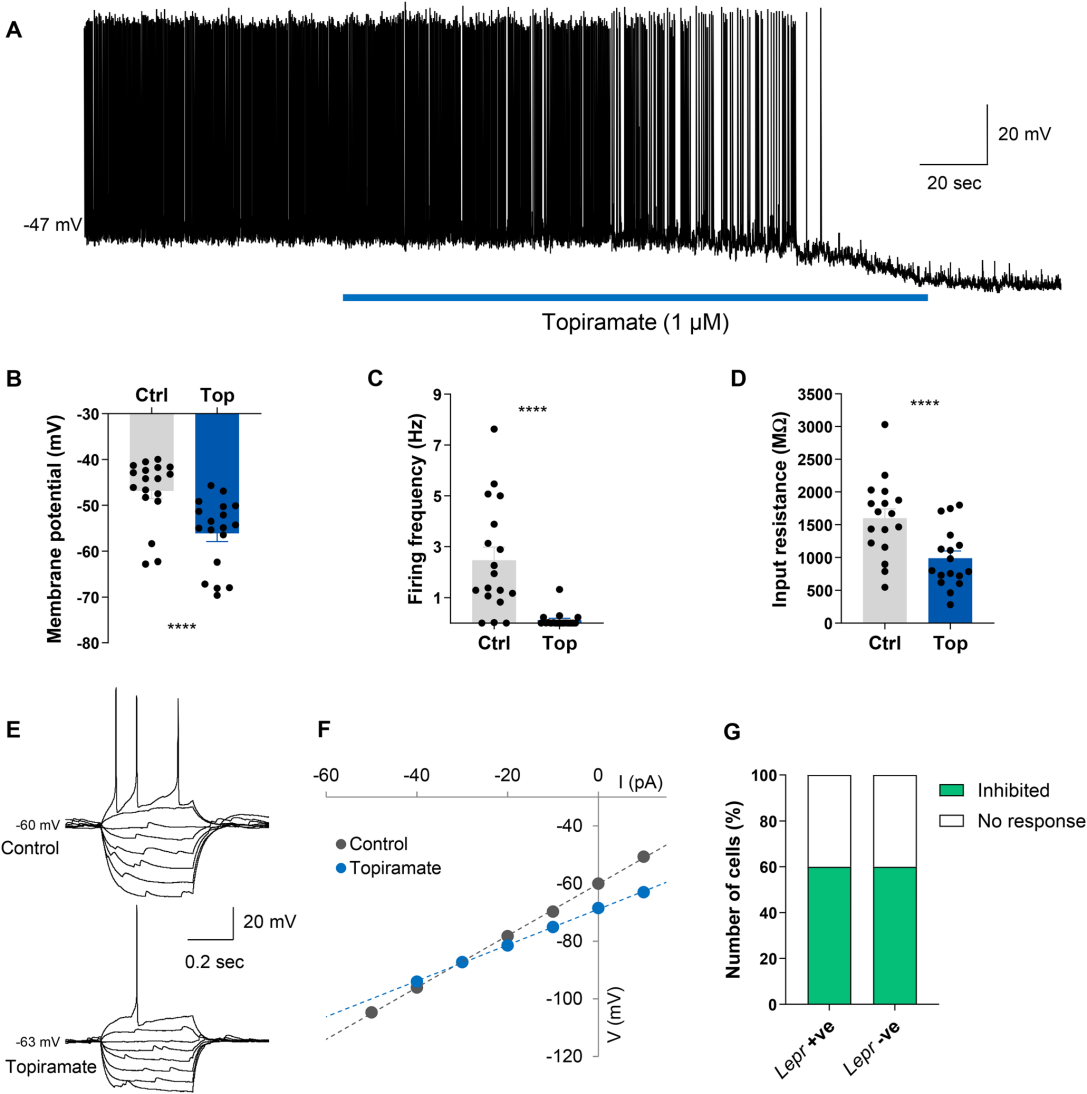
Topiramate strongly inhibits ARC NPY/AgRP neurons. (A) Current clamp recording demonstrating the inhibitory effect of topiramate on a NPY/AgRP neuron. The inhibitory effect of topiramate on NPY/AgRP neurons was accompanied by (B) a hyperpolarization of the membrane potential, (C) reduction of the firing rate, and (D) a decrease in input resistance. (E) Traces showing voltage response to current injections from a NPY/AgRP neuron that was inhibited by topiramate when held at approximately the same membrane potential. (F) Current–voltage relationships (IV) from the neuron shown in panel E illustrating a characteristic decrease in input resistance following topiramate administration. (G) Using *Npy*‐hrGFP::*Lepr*‐cre::tdTomato mice, we found that the sensitivity of NPY/AgRP neurons to topiramate was the same (60% inhibited) whether the neurons expressed tdTomato (*Lepr* ^+ve^) or not (*Lepr* ^−ve^). Ctrl = control; Top = topiramate. ****p < 0.0001. [Color figure can be viewed at wileyonlinelibrary.com]

To investigate the effects of topiramate on hypothalamic orexigenic neurons, we performed whole‐cell current clamp recordings in brain slices from male *Npy*‐hrGFP mice [23]. NPY/AgRP neurons were located in the ARC based on the expression of GFP. Bath application of topiramate (1 μM) inhibited a majority (60%; 18/30) of the NPY/AgRP neurons tested (Figure 1A). This inhibition was characterized by a significant hyperpolarization of the membrane potential from a resting potential of −46.8 ± 1.7 mV in control conditions to −56.1 ± 1.8 mV following the administration of topiramate (Wilcoxon matched‐pairs signed rank test, *W* = −171.0, *n* = 18, *p* < 0.0001) and was associated with a significant decrease in firing frequency (control: 2.47 ± 0.51 Hz vs. topiramate: 0.12 ± 0.07 Hz) (Wilcoxon matched‐pairs signed rank test, *W* = −136.0, *n* = 18, *p* < 0.0001) (Figure 1B,C). Neuronal input resistance was also assessed in 17 of the topiramate‐inhibited NPY/AgRP neurons. The inhibitory effect of topiramate was associated with a significant decrease in input resistance (control: 1597 ± 145 MΩ vs. topiramate: 987 ± 110 MΩ) (paired *t*‐test, *t*(16) = 5.887, *n* = 17, *p* < 0.0001) and a reversal potential of −79.7 ± 4.2 mV (Figure 1D–F). These data suggest that the opening of either a chloride or potassium channel may mediate the inhibitory effects of topiramate on NPY/AgRP neurons.

In a subset of the recordings, the NPY/AgRP neurons tested (*n* = 20) were from *Npy*‐hrGFP::*Lepr*‐Cre::tdTomato mice, allowing us to distinguish between *Lepr*‐Cre positive and *Lepr*‐Cre negative NPY/AgRP neurons [27]. Of these NPY/AgRP neurons, we found that 75% (15/20 cells) expressed *Lepr* (tdTomato^+^). Moreover, when we examined the response of these cells to topiramate, we found that topiramate inhibited equal proportions (60%) of *Lepr*‐positive or *Lepr*‐negative cells (Figure 1G). Despite the low numbers of *Lepr*‐negative cells recorded, these results suggest that *Lepr* expression does not associate with the sensitivity of NPY/AgRP neurons to topiramate.

The concentration of topiramate used in this study (1 μM) was estimated to fall within the clinical range. Prior work indicates that high doses of topiramate, known to cause maximum weight loss (~200 mg/day) [11], are associated with serum levels of around 4 μg/mL [30]. Cerebrospinal fluid (CSF) levels of topiramate are 85% of serum levels [31], equaling approximately 10 μM with these high doses of topiramate. However, the weight loss effects of topiramate plateau at considerably lower doses (approx. 100 mg/day) [11], suggesting that topiramate CSF levels are likely much lower in patients taking topiramate for the treatment of obesity. Nevertheless, we explored higher (10 μM) and lower (100 nM) concentrations of topiramate on NPY/AgRP neuron activity. Topiramate at 10 μM produced similar effects as the 1 μM concentration. It inhibited a majority (58%; 7/12) of NPY/AgRP neurons and was accompanied by a hyperpolarization of the membrane potential by 10.2 ± 1.5 mV. In contrast, a lower concentration of topiramate (100 nM) only inhibited 23% of NPY/AgRP neurons (3/13 cells). Therefore, 1 μM topiramate was used in all subsequent experiments.

### 3.2 GABA_A_ Receptor Is Not Required for the Inhibitory Effects of Topiramate on NPY/AgRP Neurons

**FIGURE 2:**
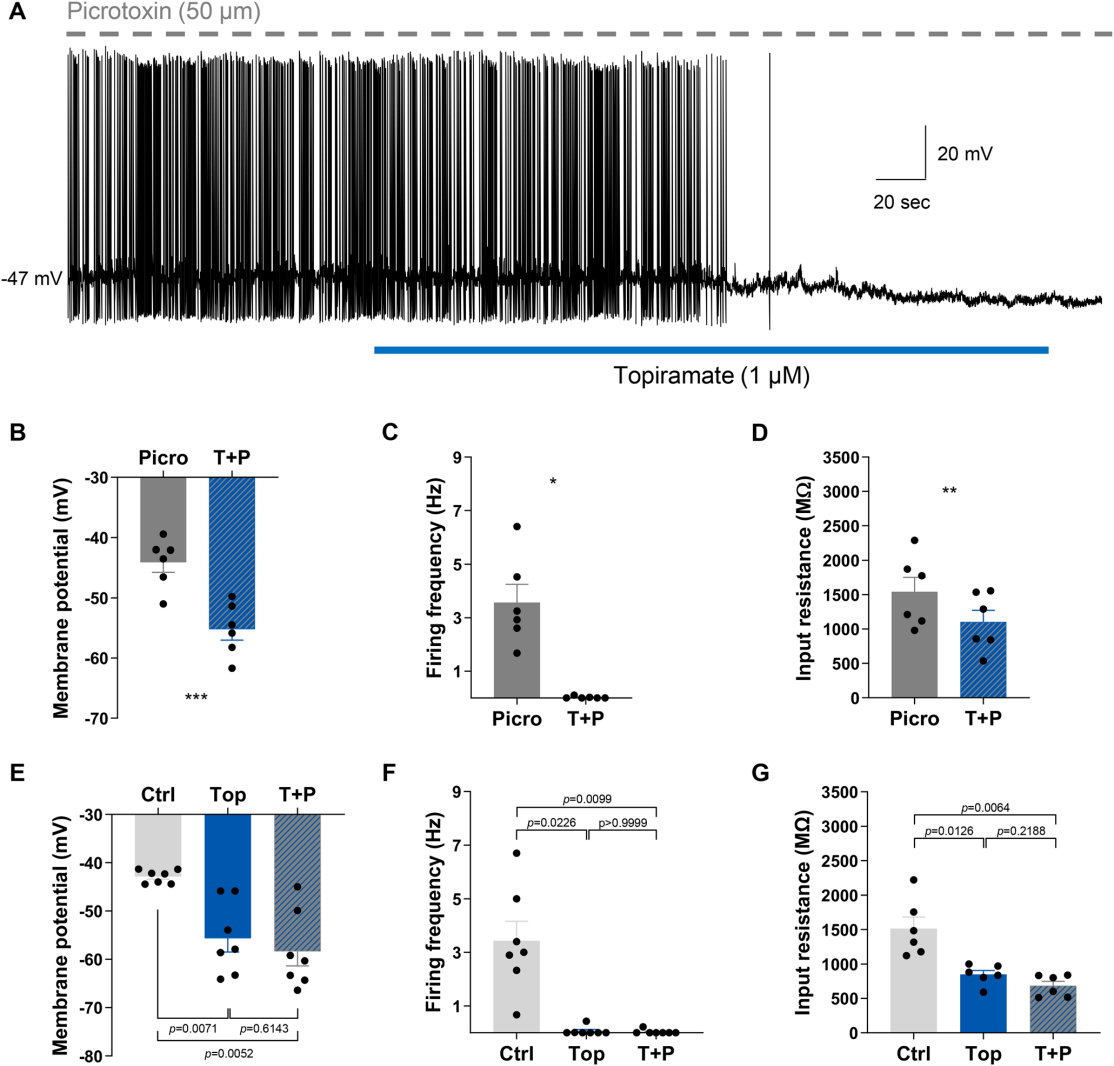
Blockade of the GABA_A_R does not influence the topiramate‐induced inhibition of ARC NPY/AgRP neurons. (A) Current clamp recording showing the inhibitory effect of topiramate on ARC NPY/AgRP neuron activity remains in the presence of GABA_A_R blocker (picrotoxin). In the presence of picrotoxin, topiramate (B) hyperpolarized the membrane potential, (C) reduced the firing frequency, and (D) reduced input resistance of NPY/AgRP neurons. In addition, picrotoxin failed to reverse the topiramate‐induced effects on (E) membrane potential, (F) firing frequency, or (G) input resistance in NPY/AgRP neurons. Picro = picrotoxin, T + P = topiramate and picrotoxin, Ctrl = control, Top = topiramate. ***p < 0.001, **p < 0.01, *p < 0.05. Adjusted *p* values for multiple comparisons are indicated in panels E–G. [Color figure can be viewed at wileyonlinelibrary.com]

Topiramate has previously been shown to target GABA_A_ receptors and to enhance GABA_A_ receptor‐mediated chloride currents [15, 16]. As the reversal potential observed in our study was between that of chloride and potassium, we aimed to determine if the GABA_A_R was mediating the topiramate‐induced inhibition of NPY/AgRP neurons. In the presence of the GABA_A_R antagonist picrotoxin (50 μM), topiramate continued to inhibit 55% of NPY/AgRP neurons (6/11 cells) (Figure 2A). Under these conditions, the topiramate‐induced inhibition of NPY/AgRP neurons was associated with a significant hyperpolarization of the membrane potential (paired *t*‐test, *t*(5) = 6.940, *n* = 6, *p* = 0.0010), a significant reduction in firing frequency (Wilcoxon matched‐pairs signed rank test, *W* = −21.00, *n* = 6, *p* = 0.0312), and a significant decrease in input resistance (paired *t*‐test, *t*(5) = 6.589, *n* = 6, *p* = 0.0012) (Figure 2B–D). The inhibitory effect of topiramate following blockade of the GABA_A_R was associated with an average reversal potential of −82.8 ± 2.4 mV. Therefore, in the presence of picrotoxin, the inhibitory effects of topiramate were very similar to the topiramate‐induced inhibition of NPY/AgRP neurons in control conditions. To further explore any potential contribution of the GABA_A_R to the inhibitory effects of topiramate, we performed additional experiments on topiramate‐inhibited NPY/AgRP neurons. Once topiramate‐inhibited NPY/AgRP neurons were identified, we tried to reverse the effects with picrotoxin. Similarly, picrotoxin had no significant effect on the electrical excitability of NPY/AgRP neurons that were inhibited by topiramate (Figure 2E–G). Together, these results suggest that the GABA_A_R is not involved in the topiramate‐induced inhibition of NPY/AgRP neurons.

### 3.3 Synaptic Transmission Is Required for the Inhibitory Effects of Topiramate

**FIGURE 3:**
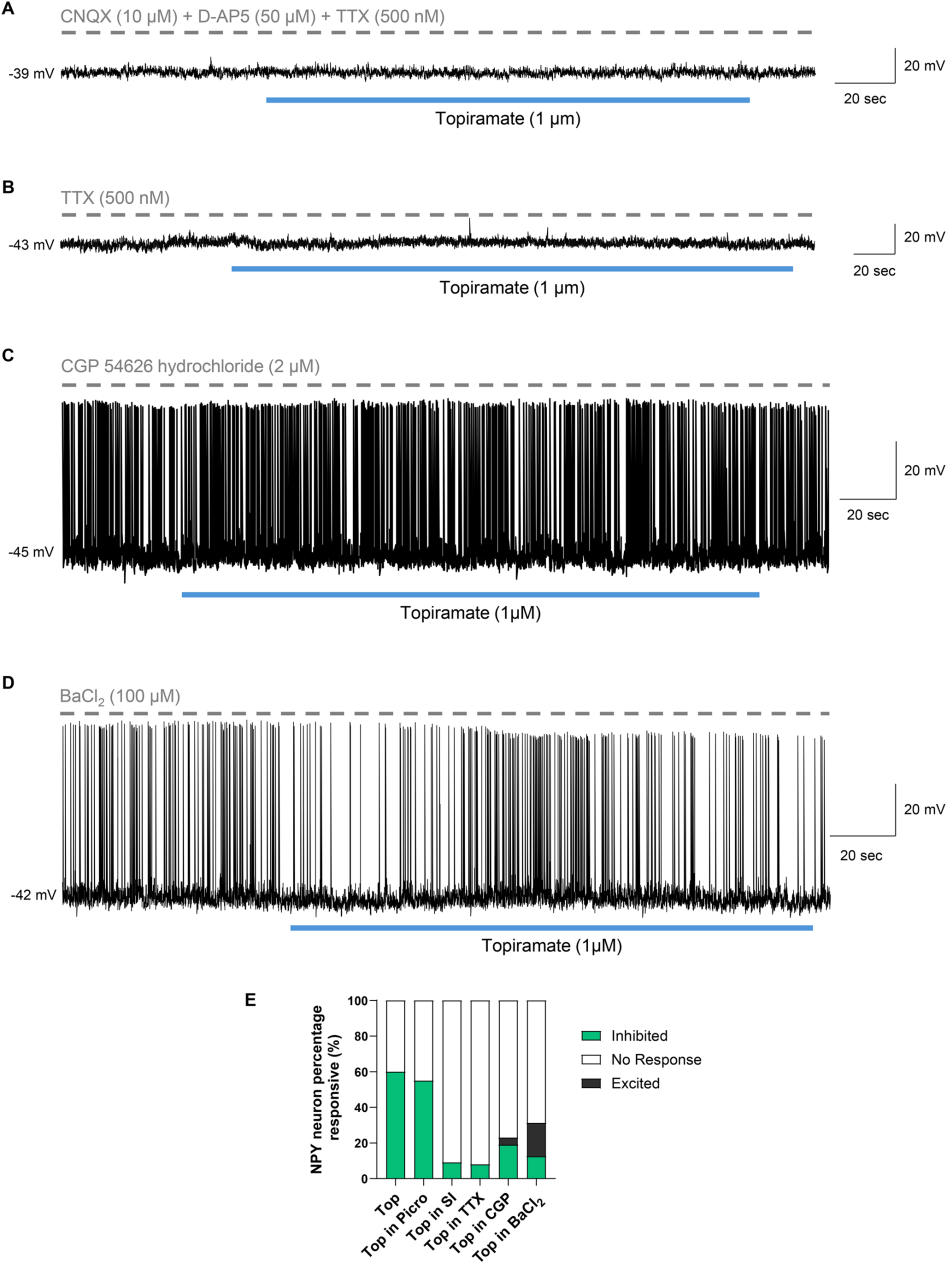
Inhibiting synaptic transmission and blockade of the GABA_B_R prevents the topiramate‐induced inhibition of ARC NPY/AgRP neurons. (A) Current clamp recording demonstrating that blockade of glutamatergic and action potential‐mediated synaptic transmission prevented the topiramate‐induced inhibition of NPY/AgRP neurons. (B) Blockade of action potential‐mediated synaptic transmission alone also prevented the topiramate‐induced inhibition of NPY/AgRP neurons. (C) Current clamp recording demonstrating that blockade of the GABA_B_R with CGP 54626 prevented the topiramate‐induced inhibition of NPY/AgRP neurons. (D) Current clamp recording demonstrating that BaCl_2_ prevented the topiramate‐induced inhibition of NPY/AgRP neurons. (E) Summary of NPY/AgRP neuron responsiveness to topiramate in the various conditions tested. The proportion of NPY/AgRP neurons inhibited by topiramate is highly suppressed after the blockade of synaptic transmission, the GABA_B_R, or inwardly rectifying potassium channels. Top = topiramate, Top in Picro = topiramate in the presence of picrotoxin, Top in SI = topiramate in the presence of synaptic inhibitors (CNQX, D‐AP5, and TTX), Top in TTX = topiramate in the presence of TTX, Top in CGP = topiramate in the presence of CGP 54626, and Top in BaCl_2_ = topiramate in the presence of barium chloride. [Color figure can be viewed at wileyonlinelibrary.com]

To determine the potential involvement of presynaptic neurons in mediating the effects of topiramate on NPY/AgRP neurons, we exposed NPY/AgRP neurons to topiramate in the presence of inhibitors of synaptic transmission. As we had already discounted the involvement of the GABA_A_R, we first used synaptic inhibitors to simultaneously block glutamatergic and action potential‐mediated synaptic transmission (CNQX, 10 μM; D‐AP5, 50 μM; and TTX, 500 nM). In the presence of these inhibitors, 91% of NPY/AgRP neurons tested (10/11 cells) failed to respond to topiramate (Figure 3A) and displayed no change in membrane potential (Wilcoxon matched‐pairs signed rank test, *W* = 27.00, *n* = 10, *p* = 0.1934) or input resistance (paired *t*‐test, *t*(9) = 1.936, *n* = 10, *p* = 0.0849). As the reversal potential of our original topiramate‐induced inhibition suggested that a glutamatergic mechanism was unlikely, we repeated these studies in the presence of TTX alone. Similarly, once action potential‐mediated synaptic transmission was blocked with TTX, 92% of NPY/AgRP neurons tested (12/13 cells) showed no response to topiramate (Figure 3B), which was associated with no change in membrane potential (paired *t*‐test, *t*(11) = 0.7779, *n* = 12, *p* = 0.4531) or input resistance (paired *t*‐test, *t*(11) = 1.699, *n* = 12, *p* = 0.1173). These results suggest that the inhibitory effects of topiramate on NPY/AgRP neuron activity require action potential‐dependent transmitter release and are likely mediated by a presynaptic mechanism.

### 3.4 Blockade of the GABA_B_ Receptor Highly Suppresses the Topiramate‐Induced Inhibition of NPY/AgRP Neurons

Given that our data suggested that the topiramate‐induced inhibition of NPY/AgRP neurons required action potential‐dependent transmitter release, was likely mediated by the opening of a potassium or chloride channel, and did not involve the GABA_A_R, we investigated if the effect could be mediated via the metabotropic GABA_B_R which can hyperpolarize neurons through the opening of potassium channels. To block the GABA_B_R, we used the potent and selective GABA_B_R antagonist (CGP 54626 hydrochloride, 2 μM). In the presence of CGP 54626, the majority (77%) of NPY/AgRP neurons (20/26 cells) were unresponsive to topiramate (Figure 3C) and displayed no change in resting membrane potential (paired *t*‐test, *t*(19) = 0.3763, *n* = 20, *p* = 0.7109) or firing frequency (Wilcoxon matched‐pairs signed rank test, *W* = −16.00, *n* = 20, *p* = 0.3125), as well as a negligible decrease in input resistance (CGP 54626: 1202 ± 162 MΩ vs. topiramate: 1182 ± 162 MΩ) (paired *t*‐test, *t*(18) = 2.230, *n* = 19, *p* = 0.0387).

As the GABA_B_R can influence G protein‐coupled inwardly rectifying potassium (GIRK) channels to initiate membrane hyperpolarization, we exposed NPY/AgRP neurons to topiramate in the presence of the Kir channel blocker Ba^2+^ (BaCl_2_: 100 μM). Under these conditions, the majority (69%) of NPY/AgRP neurons (11/16 cells) were unresponsive to topiramate (Figure 3D), with no change in resting membrane potential (paired *t*‐test, *t*(10) = 1.418, *n* = 11, *p* = 0.1865), firing frequency (Wilcoxon matched‐pairs signed rank test, *W* = −15.00, *n* = 11, *p* = 0.0625), or input resistance (paired *t*‐test, *t*(10) = 1.221, *n* = 11, *p* = 0.2500). Together, when the GABA_B_R or Kir channels were blocked, only 19% (5/26 cells) or 13% (2/16 cells) of NPY/AgRP neurons, respectively, were inhibited by topiramate, in contrast to the 60% seen with topiramate alone (Figure 3E). Therefore, these results suggest that the GABA_B_R mediates most of the inhibitory actions of topiramate on NPY/AgRP neuron activity.

### 3.5 Topiramate Does Not Influence POMC Neuron Activity

**FIGURE 4:**
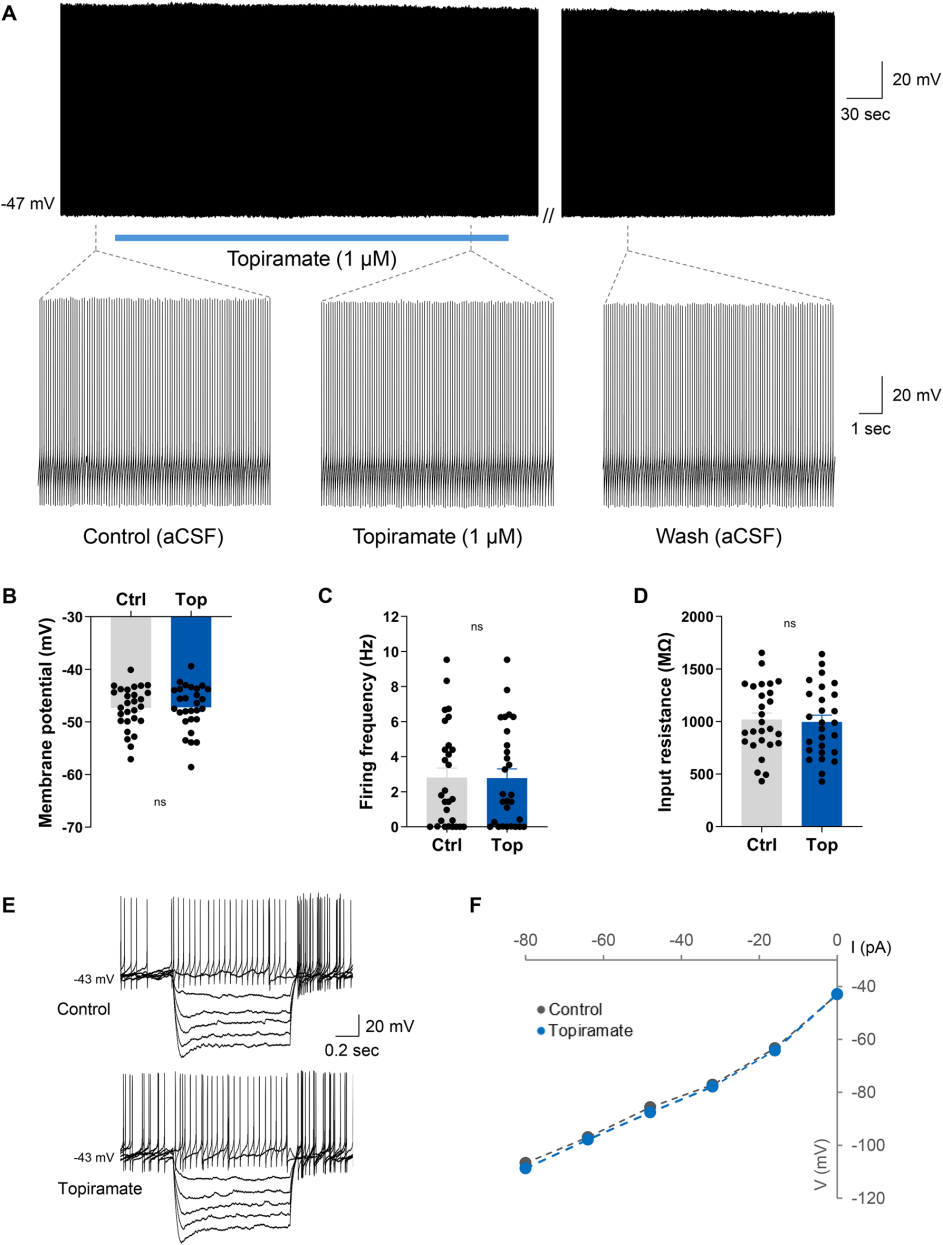
Topiramate does not influence POMC neuron electrical activity. (A) Current clamp recording demonstrating a POMC neuron that displayed no change in activity in response to topiramate, including shorter time scale segments of the recording demonstrating consistent action potential firing. Overall, topiramate did not influence POMC neuron (B) membrane potential, (C) firing frequency, or (D) input resistance. (E) Traces showing voltage response to current injections from a POMC neuron that was unresponsive to topiramate. (F) Current–voltage relationships (IV) from neuron shown in panel E illustrating no change in input resistance following topiramate administration. Ctrl = control; ns = nonsignificant; Top = topiramate. [Color figure can be viewed at wileyonlinelibrary.com]

Given that NPY/AgRP neurons represent the orexigenic component of the melanocortin system, we next examined whether topiramate influences the electrical excitability of the anorexigenic POMC neurons. Genetically identified POMC neurons in the ARC were targeted for whole‐cell current clamp recordings using male *Pomc*‐CreERT2 mice [28]. Interestingly, bath application of topiramate (1 μM) had no measurable effect on the majority of the POMC neurons tested, with 93% (28/30 cells) showing no significant change in electrical properties (Figure 4A). In these cells, topiramate failed to influence resting membrane potential (paired *t*‐test, *t*(27) = 0.7163, *n* = 28, *p* = 0.4799), firing frequency (Wilcoxon matched‐pairs signed rank test, *W* = −29.00, *n* = 28, *p* = 0.4294), or input resistance (paired *t*‐test, *t*(26) = 1.548, *n* = 27, *p* = 0.1338) in those cells in which it was measured (Figure 4B–F). A minor inhibitory effect was observed in the remaining two POMC neurons tested (7%), which exhibited a modest hyperpolarization of the membrane potential (−5.2 ± 0.3 mV). However, these effects were rare and likely not representative of the population as a whole. These results indicate that topiramate does not significantly alter the excitability of POMC neurons.

### 3.6 Melanocortin Neurons From Female Mice Display Similar Responses to Topiramate as Observed in Males

**FIGURE 5:**
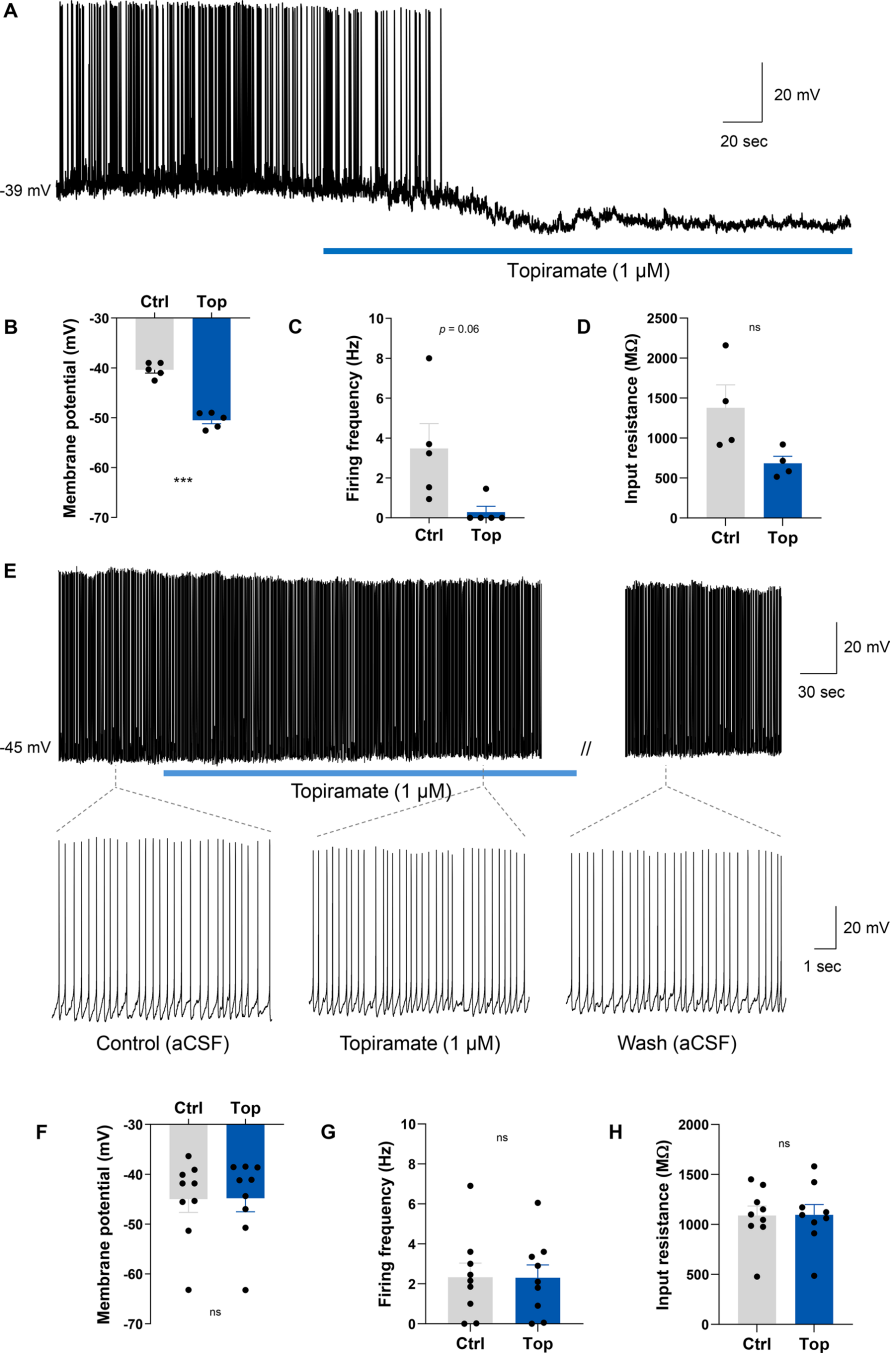
Topiramate influences NPY/AgRP but not POMC neuron activity in female mice, similar to males. (A) Current clamp recording demonstrating the inhibitory effect of topiramate on a NPY/AgRP neuron obtained from a female mouse. The inhibitory effect of topiramate on NPY/AgRP neurons from female mice was accompanied by (B) a hyperpolarization of the membrane potential, (C) a reduction of the firing rate, and (D) a decrease in input resistance. (E) Current clamp recording demonstrating a POMC neuron obtained from a female mouse that displayed no change in activity in response to topiramate, including shorter time scale segments of the recording demonstrating consistent action potential firing. Overall, topiramate did not influence POMC neuron (F) membrane potential, (G) firing frequency, or (H) input resistance. Ctrl = control, Top = topiramate. ***p < 0.001, ns = nonsignificant. [Color figure can be viewed at wileyonlinelibrary.com]

To determine whether the effects of topiramate on melanocortin neurons are influenced by sex, we repeated our experiments in female *Npy*‐hrGFP and *Pomc*‐CreERT2 mice. Similar to males, 50% of NPY/AgRP neurons (5/10 cells) from female mice were inhibited by topiramate. The inhibitory effect was associated with a significant hyperpolarization of the membrane potential from a resting potential of −40.4 ± 0.7 to −50.5 ± 0.7 mV following the administration of topiramate (paired *t*‐test, *t*(4) = 13.22, *n* = 5, *p* = 0.0002) and a trend towards a decreased firing rate (Wilcoxon matched‐pairs signed rank test, *W* = −15.00, *n* = 5, *p* = 0.0625). Input resistance was measured in 4 of these cells, and although it decreased, this effect was not statistically significant (paired *t*‐test, *t*(3) = 2.152, *n* = 4, *p* = 0.1205) (Figure 5A–D).

In POMC neurons from female mice, topiramate had little to no effect, mirroring results in males: 90% of cells (9/10) were unresponsive. These neurons displayed no difference in resting membrane potential (Wilcoxon matched‐pairs signed rank test, *W* = 11.00, *n* = 9, *p* = 0.5703), firing frequency (paired *t*‐test, *t*(8) = 0.2017, *n* = 9, *p* = 0.8452), or input resistance (paired *t*‐test, *t*(8) = 0.2860, *n* = 9, *p* = 0.7821) between control and topiramate conditions (Figure 5E–H). These results closely match those obtained in males, indicating that the effects of topiramate on melanocortin neuron activity are not sex‐dependent.

**FIGURE 6:**
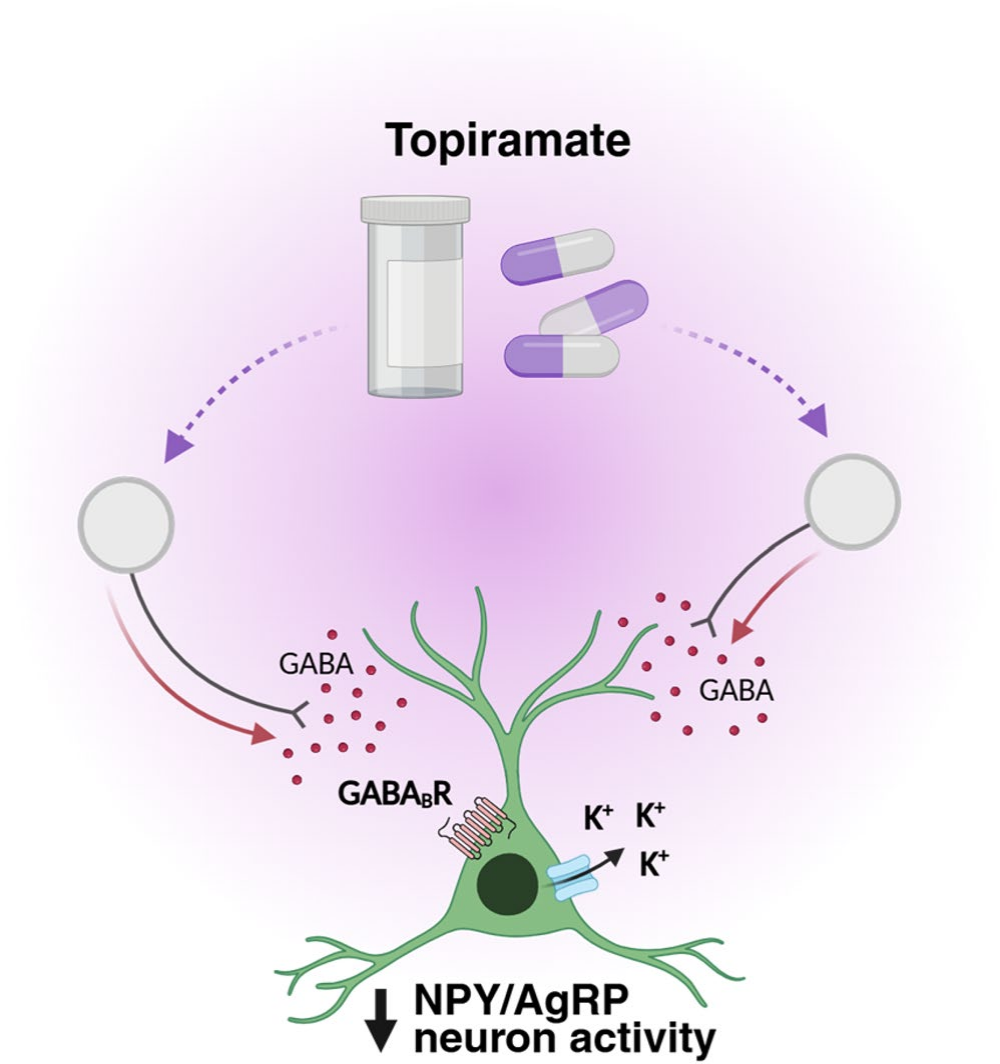
Working model of the action of topiramate on NPY/AgRP neurons. Topiramate increases GABAergic tone to ARC NPY/AgRP neurons, thereby inhibiting their activity via activation of the GABA_B_R. Figure created in BioRender. Michael (2025) https://BioRender.com/juolv2f. [Color figure can be viewed at wileyonlinelibrary.com]

Collectively, these results indicate that topiramate does not significantly alter the excitability of POMC neurons and suggest that topiramate's actions within the melanocortin system are primarily mediated through inhibition of NPY/AgRP neuron activity (Figure 6).

## 4 Discussion

In this study, we investigated whether the weight‐lowering medication topiramate influences the activity of key hypothalamic neurons involved in energy balance regulation. We demonstrate that topiramate exerts a strong inhibitory effect on orexigenic NPY/AgRP ARC neurons. This inhibitory effect was highly reduced when action potential‐dependent synaptic transmission was blocked and when GABA_B_ receptors were antagonized, suggesting that topiramate may act at presynaptic neurons to modify GABAergic neurotransmission to the NPY/AgRP neuron synapse, with subsequent activation of postsynaptic GABA_B_ receptors and potassium channels. While our data support a model in which GABA_B_ receptors expressed on NPY/AgRP neurons mediate the inhibitory effects of topiramate, additional contributions from presynaptic GABA_B_ receptors cannot be excluded. Notably, topiramate had minimal impact on the excitability of anorexigenic POMC neurons, highlighting selectivity within the melanocortin system. Together, these findings identify NPY/AgRP neurons as key targets of topiramate and suggest that enhanced GABAergic tone to these neurons contributes, at least in part, to topiramate's weight‐lowering effects (Figure 6).

Although the topiramate‐induced inhibition of NPY/AgRP neurons was sensitive to TTX, indicating a requirement for action potential‐dependent synaptic transmission, it remains unclear if, and how, topiramate activates presynaptic GABAergic neurons. NPY/AgRP neurons receive GABAergic inputs from both within and outside of the ARC. Within the ARC, these include thyrotropin‐releasing hormone (TRH)‐expressing [26] and basonuclin 2 (BNC2)‐expressing neurons [25], while long‐range GABAergic projections arise from the DMH, including populations expressing LepR or glucagon‐like peptide‐1 receptor (GLP‐1R) [24, 32]. These inputs suppress NPY/AgRP neuron activity, promote satiety, and inhibit food intake [24, 25, 26, 32]. As such, they represent potential targets of topiramate action that, upon activation, could account for the observed inhibition of NPY/AgRP neurons. Additional candidates include ARC GABAergic neurons expressing cellular retinoic acid binding protein 1 (CRABP1), which are sensitive to metabolic status and may directly innervate AgRP neurons [26, 33]. Future studies will be required to determine whether topiramate selectively engages one or more of these presynaptic populations to enhance GABAergic tone and suppress orexigenic signaling.

Hypothalamic NPY/AgRP and POMC neurons are well‐established regulators of energy homeostasis with opposing actions on downstream targets [19]. Accordingly, most metabolic cues and AOMs have traditionally been shown to influence both populations in opposing directions to achieve weight loss [20, 34, 35]. Therefore, our findings that topiramate selectively inhibits NPY/AgRP neurons without affecting POMC neurons were unexpected. However, recent advances in neurocircuit mapping have revealed considerable complexity in the hypothalamic feeding circuits. These include the identification of novel neuronal populations and distinct mechanisms by which metabolic signals such as leptin and GLP‐1 regulate energy balance. Importantly, accumulating evidence supports the existence of segregated GABAergic inputs that differentially regulate NPY/AgRP and POMC neurons [24, 36]. Therefore, it is plausible that topiramate selectively modulates presynaptic inputs to NPY/AgRP and not POMC neurons, consistent with our observations. Additionally, since NPY/AgRP neurons can directly inhibit POMC neurons [19], the inhibitory effects of topiramate on NPY/AgRP neurons could reasonably lead to disinhibition of POMC neurons. However, recent work suggests that NPY/AgRP‐derived inhibitory inputs account for only a minor fraction of the spontaneous inhibitory tone onto POMC neurons [18]. In line with this, we observed no measurable effect of topiramate on POMC neuron activity.

The potential for inhibitory actions of topiramate on NPY/AgRP neurons has previously been assumed to occur via actions at GABA_A_ receptors [2]. However, our data suggest that activation of potassium channels, likely through GABA_B_ receptor activation and not GABA_A_ receptors, mediates the topiramate‐induced inhibition of NPY/AgRP neurons. Such effects are consistent with reports that topiramate increases brain GABA levels and can activate potassium channels [17, 37]. Interestingly, this mechanism differs from other AOMs. For instance, liraglutide, a GLP‐1R agonist, inhibits NPY/AgRP through presynaptic mechanisms and activates POMC neurons through both pre‐ and postsynaptic mechanisms [20]. In contrast to liraglutide, which inhibits NPY/AgRP neurons via GABA_A_ receptors, here we demonstrate that topiramate inhibits NPY/AgRP neurons independently of GABA_A_ receptors. Our work suggests that GABA_B_ receptors play a major role in the topiramate‐induced inhibition of NPY/AgRP neurons. Multiple lines of evidence suggest that NPY/AgRP neurons may be heterogeneous [38, 39], and single cell sequencing strongly supports this concept [40]. Importantly, such work has demonstrated that not all ARC NPY neurons express AgRP [39, 40], and that the NPY neurons that coexpress AgRP mainly express GABA_B_ receptors, whereas the population only expressing NPY expresses subunits for both GABA_A_ and GABA_B_ receptors [40]. Such heterogeneity may explain how AOMs selectively modulate these cells through distinct mechanisms. These different mechanisms of action also justify testing topiramate in combination with other AOMs for potential additive effects, although such a possibility would need to be assessed.

Topiramate is currently approved for obesity treatment in combination with phentermine. While average weight loss with this combination is around 10% [41, 42], some individuals can achieve reductions of 15%–20% or more [43]. Notably, phenotype‐guided treatment approaches have been associated with enhanced weight loss outcomes when topiramate/phentermine is matched to specific patient profiles [44]. Moreover, the lower cost of topiramate/phentermine compared to some other AOMs makes it a more accessible option across diverse socioeconomic groups [45]. Therefore, despite the emergence of newer classes of AOMs, topiramate/phentermine is a highly effective option in certain patient populations, supporting the importance of understanding its neurobiological mechanisms of action. While the present study focused on the effects of topiramate on melanocortin neuron activity under chow‐fed conditions, future work should examine whether these actions are preserved or altered in models of obesity.

In summary, we provided the first demonstration that topiramate strongly inhibits hypothalamic NPY/AgRP neuron activity. Our data also suggest that enhanced GABAergic tone mediates the effect. The inhibitory actions of topiramate on NPY/AgRP neurons likely contribute to its appetite‐suppressing and weight‐reducing effects. While the metabolic benefits of topiramate have been well documented, its mechanisms of action in the brain have remained elusive. These findings may inform future efforts to optimize or combine topiramate with other AOMs to maximize therapeutic benefit.

## Author Contributions

Conceptualization: K.W.W. and N.J.M. Methodology: K.W.W. and N.J.M. Investigation: M.M.M., O.L., A.C., and N.J.M. Formal analysis: M.M.M., O.L., and N.J.M. Writing – original draft: M.M.M. and N.J.M. Writing – review and editing: M.M.M., O.L., A.C., K.W.W., and N.J.M. Funding acquisition: N.J.M. Supervision: A.C. and N.J.M. All authors contributed to editing the manuscript and approved the manuscript for publication.

## Conflicts of Interest

The authors declare no conflicts of interest.

## Data Availability

The data that support the findings of this study are available from the corresponding author upon reasonable request.
